# Surgical Resection of a Giant Hepatocellular Carcinoma in an 89-Year-Old Patient with Multiple Comorbidities: The Role of Frailty Assessment and Preoperative Optimisation—A Case Report and Literature Review

**DOI:** 10.3390/diagnostics16121902

**Published:** 2026-06-19

**Authors:** Milan Krnojelac, Lea Andjelković

**Affiliations:** 1 The Division of Internal Medicine, University Medical Centre Ljubljana, 1000 Ljubljana, Slovenia; milan.krnojelac@kclj.si; 2 Clinical Department of Anaesthesiology and Intensive Therapy, University Medical Centre Ljubljana, 1000 Ljubljana, Slovenia; 3 Department of Anesthesiology and Reanimation, University of Ljubljana, 1000 Ljubljana, Slovenia

**Keywords:** hepatectomy, carcinoma, hepatocellular, frailty, perioperative care, comorbidity, multidisciplinary team

## Abstract

**Background and Clinical Significance:** Liver cancer incidence increases with age, and surgical resection remains the primary curative option for early-to-intermediate hepatocellular carcinoma. In elderly patients, multimorbidity and frailty complicate risk assessment, so selection should consider more than chronological age. **Case Presentation:** An 89-year-old woman with hypertension, heart failure, atrial fibrillation on anticoagulation, and significant proximal LAD stenosis underwent preoperative percutaneous revascularisation and a left lateral sectionectomy for a solitary liver mass. Histology confirmed hepatocellular carcinoma; the early postoperative course required brief ICU support for haemodynamic instability and supplemental oxygen, with discharge on postoperative day six. **Conclusions:** This case report shows that, after multidisciplinary evaluation and targeted preoperative optimisation (including cardiac intervention), selected very elderly patients with multiple comorbidities may be considered for liver resection, though they remain at increased risk of perioperative cardiopulmonary and haemodynamic events. Incorporating frailty, nutritional, and functional assessments alongside comorbidity profiling improves patient selection and supports individualised decision-making.

## 1. Introduction

Liver cancer is the third most common cause of cancer-related death worldwide. Incidence is highest in Asia and increases with patient age [1]. Life expectancy has also risen globally, particularly in developed regions. Data from 2023 indicate that life expectancy at birth in the EU was 81.5 years [2]. Consequently, more elderly patients with liver malignancies are expected. Surgical resection is regarded as the gold standard for curative treatment of early-to-intermediate-stage hepatocellular carcinoma, the most common type of liver malignancy [3]. In the literature, patient age is identified as a risk factor for complications and mortality in liver surgery. In elderly patients, treatment selection is complicated by comorbidities, increased surgical risks, and a lack of objective data. Many studies also exclude patients aged 80 or above, reducing the reliability of data. Therefore, guidelines are needed that account for all factors affecting the success rate of surgical procedures in the elderly [4]. The WHO defines elderly or older adults as persons aged 60 or above. Many clinical studies divide patients into two groups: above and below 60 or 65 years of age. A systematic review and meta-analysis show that 68.5 years is the calculated age after which postoperative morbidity and mortality increase, mostly due to age-related comorbidities such as cardiovascular diseases [5].

This article presents a challenging case of hepatocellular carcinoma in an 89-year-old woman with multiple comorbidities and haemodynamically significant coronary artery stenosis. The case demonstrates that, in carefully selected very elderly patients, targeted preoperative cardiac intervention may expand surgical candidacy even when multiple comorbidities are present.

This article includes a case report and a focused narrative review of the literature. Relevant publications were identified through structured searches of PubMed and related biomedical databases from 1995 to 2025, using combinations of the following keywords: liver resection, hepatocellular carcinoma, elderly patients, ageing, frailty, comorbidity, perioperative risk, surgical outcomes, and hepatectomy. Inclusion criteria were peer-reviewed studies published in English between 1995 and 2025 reporting postoperative outcomes in elderly patients (aged ≥ 60 years) undergoing liver resection. Studies were excluded if they did not report age-stratified outcomes, were published in languages other than English, or were case reports other than the present one.

Priority was given to systematic reviews, meta-analyses, large cohort studies, and international clinical guidelines from major hepatobiliary and surgical societies. Additional emphasis was placed on studies examining surgical outcomes in elderly patients, particularly those evaluating frailty, functional status, and comorbidity burden.

Publications were selected for their relevance to clinical decision-making in elderly patients undergoing liver resection, with particular focus on factors beyond chronological age and on multidisciplinary perioperative management. The selected literature was used to contextualise and support the findings of the case presented.

## 2. Case Report

An 89-year-old woman was treated at the Department of Abdominal Surgery, University Medical Centre Ljubljana, for a solitary mass in the third liver segment. A left lateral sectionectomy was performed.

### 2.1. Presentation and Preoperative Assessment

The patient had a history of arterial hypertension, heart failure, atrial fibrillation on anticoagulant therapy, hyperlipidaemia, osteoporosis, urinary incontinence, and urinary bladder prolapse.

In May 2024, the patient presented with abdominal pain below the right costal margin. Clinical examination revealed no abnormalities. Abdominal ultrasound showed a 47 mm heterogeneous, well-demarcated tumour in the left liver lobe. Initial laboratory results are shown in Table 1. A contrast-enhanced CT performed in September 2024 described the lesion as having a diameter of 6.1 cm, and AFP was 26 IU/mL at that time (Figure 1 and Figure 2).

In September 2024, the patient underwent a liver MRI. In the left liver lobe, an oval, well-demarcated, heterogeneous tumour was identified, measuring 8.4 × 6.8 × 7.5 cm. The radiologist concluded that the lesion did not display uniform characteristics typical of a specific hepatic lesion. It may represent an atypical focal nodular hyperplasia (FNH), which would be unusual; a hepatic adenoma, which would be atypical for this age group; or fibrolamellar hepatocellular carcinoma (HCC), which would also be unusual for this age. Follow-up was recommended, which may be performed with ultrasound, with correlation to laboratory parameters of alpha-fetoprotein.

In December 2024, at the gastroenterology department, alpha-fetoprotein levels were 34 IU/mL. The patient had no clinical signs of malignancy. The only symptom was palpatory pain in the upper abdomen. According to the radiological assessment, considering multiple comorbidities, advanced age, performance status, and the patient’s wish not to pursue further evaluation of the lesion, it was concluded that targeted intervention was not indicated.

In July 2025, the patient was examined at the General Hospital Jesenice because of three months of persistent nausea and vomiting after meals, loss of appetite, and weight loss. In the past six months, she had lost 25 kg. Other clinical findings were unchanged.

Laboratory results are shown in Table 1.

CT imaging of the abdomen showed an enlarged liver with a diameter of 20 cm in the midclavicular line, due to a large tumour in the third liver segment (dimensions: 11.8 × 12.3 cm) (Figure 3 and Figure 4).

Between December 2024 and July 2025, the patient showed clear clinical and radiological deterioration. The tumour grew rapidly from 6.1 cm to 11.8 × 12.3 cm, AFP rose markedly from 34 to 395.5 IU/mL, and there was significant weight loss together with new gastrointestinal symptoms. This represented a clear change in the clinical picture and prompted reassessment by the multidisciplinary team.

The multidisciplinary medical team for hepatobiliary diseases, after reviewing the medical documentation, recommended a left lateral sectionectomy. Preoperatively, the patient underwent screening with validated frailty tools. The modified Frailty Index-5 (mFI-5) was 3, placing the patient in the high-risk frail category. On the Geriatric 8 (G8) tool the score was 6 of 17—well under the cut-off of 14—which prompted referral for comprehensive geriatric assessment. After reviewing these scores together with the clinical and radiological findings, the multidisciplinary team decided to proceed with surgery once targeted preoperative optimisation had been completed. It should be noted that formal nutritional screening using standardised tools such as NRS-2002 or MUST was not documented as part of the preoperative workup, and sarcopenia was not formally assessed. Nutritional compromise was nonetheless clinically evident from the degree of weight loss, preoperative albumin of 37 g/L, and the nutritional domain of the G8 score. Liver function was evaluated using validated scoring systems prior to surgery. The patient had no history of cirrhosis, portal hypertension, or hepatic encephalopathy, and there was no clinical or imaging evidence of ascites. Preoperative laboratory values—total bilirubin 17 µmol/L, albumin 37 g/L, and INR 1.0—yielded a Child–Pugh score of 5 (class A), a MELD score of 11, and an ALBI score of −2.33 (grade 2), all consistent with well-preserved hepatic synthetic function despite the presence of a large hepatic tumour. As the patient had no underlying parenchymal liver disease, left lateral sectionectomy was expected to leave an adequate future liver remnant. Following anaesthesiological assessment, a cardiac CT was performed due to suspected angina pectoris at rest. Because of pronounced calcification of the coronary arteries and resulting artefacts, reliable assessment of the degree of coronary artery stenosis was not possible (Figure 5). The cardiologist recommended coronary angiography in the catheter laboratory. Percutaneous coronary intervention was performed on 28 August 2025. Haemodynamically significant stenosis of the proximal LAD was identified. In the other vessels (LCA, LCX, RCA), borderline changes were observed (Figure 6). Percutaneous revascularisation without stenting was performed before the planned surgery, as it required a shorter course of antiplatelet therapy: acetylsalicylic acid 100 mg for one week and clopidogrel 75 mg intended for one month, with cangrelor as perioperative bridging. Warfarin was withheld before the intervention and restarted postoperatively, at which point antiplatelet therapy was stopped on the cardiology team’s advice.

Echocardiography performed in 2018 had documented normal left ventricular systolic function and mild-to-moderate pulmonary hypertension. No repeat echocardiographic assessment was performed as part of the current preoperative workup.

### 2.2. Operative Details

A left lateral sectionectomy was performed on 4 September 2025 via an open approach. Operative time was approximately 180 min, with estimated blood loss of 300 mL. No intraoperative complications were recorded. Lesional tissue from segments II and III was pathologically confirmed as hepatocellular carcinoma. Parenchymal transection was performed with stapling devices and selective vascular ligation. A CH21 subphrenic drain was placed.

### 2.3. Pathology

Histopathological examination of the resected specimen confirmed multifocal hepatocellular carcinoma of a pseudoglandular and trabecular pattern, moderately differentiated. The largest tumour diameter measured 11 cm. Microvascular invasion was suspected. The tumour was confined to segments II–III; surgical margins were negative, confirming an R0 resection, and the pathological stage was pT3.

### 2.4. Early Postoperative Course and Short-Term Follow-Up

On the day of surgery, the patient was admitted to the intensive care unit due to haemodynamic instability. Noradrenaline was administered for vasoactive support, and supplemental oxygen was required. Vasopressor support and supplemental oxygen were both discontinued on postoperative day three. A pleural effusion of at least 500 mL was identified on ultrasound during the ICU stay and was managed with intravenous furosemide, resolving prior to ICU discharge. On postoperative day four, the patient was transferred to the surgical ward. Traces of fluid, up to 100 mL, were noted under the liver, around the spleen, and in the upper part of the intestine, consistent with expected post-hepatectomy findings. At the resection margin, a 7 × 4 × 2 cm heterogeneous formation was identified, consistent with a postoperative haematoma, a recognised finding following open liver resection. In segment IV of the liver, five round hypoechogenic formations were found, which were suspected to be secondary deposits. These findings were discussed at the postoperative multidisciplinary team meeting. As no preoperative imaging had identified lesions in segment IV, and given the patient’s clinical condition at that time, further cross-sectional imaging was not performed and their nature could not be confirmed. This represents a limitation of the present case. The findings contributed to the decision to recommend supportive rather than additional oncological treatment. On the sixth postoperative day, the patient was discharged to home care. Laboratory results are shown in Table 1.

At the outpatient visit on 29 September 2025, the patient reported relative well-being. A small superficial wound dehiscence was noted and treated with local wound care, and oral antibiotics were prescribed for a local infection. The hypoechogenic lesions seen in segment IV on the postoperative ultrasound were also discussed at this visit. Because of the patient’s advanced age, frailty and substantial comorbidity, and in line with the multidisciplinary team’s postoperative decision for supportive care, no further cross-sectional imaging was performed, as it would not have changed management. By the most recent follow-up, on 13 February 2026, the patient remained clinically compensated, with a fully healed incision and manageable constipation.

## 3. Discussion

Currently, liver resection in elderly patients remains challenging due to comorbidities and perioperative demands. The literature describes several selection approaches for liver resection in the elderly. This case of hepatic resection in an 89-year-old patient with multiple comorbidities and preoperatively treated coronary artery disease provides an opportunity to critically examine the two dominant paradigms in surgical risk stratification for elderly patients: age-based and frailty-based approaches. The following discussion contextualises the clinical decisions made and the postoperative course observed against the available evidence.

In this patient, the decision to proceed with surgery without additional MRI or preoperative biopsy was deliberate and clinically justified. The pronounced interval enlargement of the lesion, the marked rise in AFP, and the patient’s clinical deterioration collectively established a high preoperative likelihood of malignancy. During multidisciplinary review, the tumour board concluded that contrast-enhanced CT provided sufficient anatomical detail regarding tumour extent, vascular relations, and resectability to allow safe operative planning. Furthermore, delaying surgery for percutaneous biopsy was considered to carry an unacceptable risk of tumour progression and needle-tract seeding without any potential change in management. Alternative treatment modalities, including transarterial chemoembolisation (TACE) and systemic therapy, were considered but judged less appropriate in this setting. Although large and rapidly progressive, the tumour was confined to the left lateral sector, which made it anatomically suitable for resection with a negative margin. Given its size and the symptoms it was producing, removal was indicated in its own right. TACE in a lesion of this magnitude carries a substantial risk of post-embolisation syndrome and incomplete response and would not have offered curative intent. Supportive care alone was inconsistent with the patient’s clearly expressed wish for active treatment and with her otherwise preserved functional status, aside from the acute nutritional decline.

Equally important was the management of the patient’s cardiac comorbidity in the context of surgical urgency. Percutaneous coronary intervention was performed on 28 August 2025, with left lateral sectionectomy following seven days later on 4 September 2025. Balloon angioplasty without stenting was preferred over stent implantation because it requires a much shorter period of dual antiplatelet therapy. In this patient, the dual phase, comprising aspirin 100 mg together with clopidogrel 75 mg, was limited to one week, after which aspirin was stopped and clopidogrel was intended to continue alone for up to one month. Cangrelor was used as bridging therapy during the perioperative period, which allowed surgery to proceed without an excessive bleeding risk. Warfarin was withheld before the intervention and reintroduced postoperatively, and antiplatelet therapy was discontinued once warfarin had been restarted, in line with the cardiology team’s recommendation. Further delay was not a realistic option. Over 14 months the tumour had grown from 4.7 cm to 11.8 × 12.3 cm, and the AFP had risen to 395.5 IU/mL.

The choice of surgical approach in elderly patients undergoing liver resection has received increasing attention in recent studies. Multiple studies and meta-analyses have examined the comparative outcomes of open liver resection (OLR), laparoscopic liver resection (LLR), and robotic liver resection (RLR). A meta-analysis of 12 propensity score-matched studies involving 1861 patients showed that LLR was associated with significantly lower postoperative complication rates (OR 0.49, *p* < 0.00001), reduced intraoperative blood loss, and shorter hospital stay compared to OLR, with no significant differences in overall survival or disease-free survival at 1, 3, and 5 years [6]. These findings were corroborated by a systematic review of 3051 patients comparing minimally invasive liver resection (MILR) with OLR, which showed that MILR reduced blood loss by a mean of 161 mL, morbidity by 42%, and length of hospital stay by 4 days, with a non-significant trend towards reduced postoperative mortality (*p* = 0.06) [7]. A propensity score-matched analysis of 665 patients aged 70 years or older from eight European centres confirmed significantly lower rates of Clavien–Dindo grade III/IV complications in the laparoscopic group (6% vs. 20%, *p* = 0.04) and a shorter median hospital stay (5 vs. 7 days, *p* < 0.001), with comparable long-term survival [8]. Regarding robotic surgery specifically, a propensity score-matched single-centre study of 100 patients demonstrated comparable short-term outcomes and perioperative complication rates between patients aged 75 years or older and younger patients undergoing robot-assisted liver surgery [9]. When comparing LLR and RLR directly in elderly patients, a large NSQIP database analysis of 2210 patients aged 65 years or older found that RLR was associated with shorter hospital stays (3.5 vs. 4.4 days, *p* < 0.001) and longer operative times (221.4 vs. 203.5 min, *p* = 0.013), with no significant differences in serious complications or mortality on adjusted analysis [10]. A network meta-analysis of 6582 patients with colorectal liver metastases further showed that both LLR and RLR were associated with reduced postoperative complication rates compared to OLR, while RLR additionally reduced the risk of conversion to open surgery and intraoperative blood transfusions [11]. The feasibility of minimally invasive approaches in elderly patients with colorectal liver metastases was also confirmed in a study of 145 patients comparing hand-assisted laparoscopic surgery (HALS) in patients older and younger than 75 years, which demonstrated comparable complication rates and acceptable long-term outcomes in the elderly cohort [12].

Beyond the general comparison of surgical approaches, tumour size introduces an additional layer of complexity in approach selection. For large HCC (≥5 cm) in elderly patients aged 70 years or older, a multicentre propensity score-matched analysis of 363 patients showed that LLR was associated with shorter hospital stay (7 vs. 9 days, *p* = 0.01) and lower R1 resection rates (4.4% vs. 13.3%, *p* = 0.03), with no significant differences in major complications, liver failure, or survival outcomes [13]. A multicentre study of 981 patients with large (5–10 cm) or huge (>10 cm) HCC confirmed that LLR was associated with reduced operative duration, blood loss, and hospital stay, while maintaining comparable long-term survival [14]. These findings were supported by a meta-analysis of 1338 patients, which showed significantly fewer total and major postoperative complications with LLR (OR 0.49, *p* < 0.00001; OR 0.54, *p* = 0.003, respectively) and shorter hospital stay, with comparable R0 resection rates and long-term survival [15]. For truly huge HCC (≥10 cm), a propensity score-matched analysis from Eastern and Western referral centres showed that minimally invasive liver surgery reduced intraoperative blood loss (500 vs. 800 mL, *p* = 0.02), transfusion rates (25.6% vs. 48.7%, *p* = 0.03), and hospital stay (7 vs. 10 days, *p* < 0.01), with improved disease-free survival (49.8 vs. 7 months, *p* < 0.01) compared to OLR [16].

The surgical complexity of this case is further highlighted by comparative data on tumour size and resection outcomes. A large meta-analysis showed that resection of giant HCC (≥10 cm) is associated with significantly worse long-term oncological outcomes than non-giant HCC, with a hazard ratio for overall survival of 0.53 (95% CI 0.50–0.55, *p* < 0.001) and for disease-free survival of 0.62 (95% CI 0.58–0.84, *p* < 0.001), while perioperative safety profiles were comparable between the two groups [17]. Beyond this threshold, the so-called super-giant category (≥15 cm) constitutes a further distinct surgical entity with limited published data. Shelat et al. reported on 18 patients with a median tumour diameter of 172.5 mm who underwent hepatectomy, demonstrating that primary resection is technically feasible with acceptable perioperative morbidity; however, long-term outcomes were poor, with 1-, 2-, and 3-year overall survival rates of 49%, 39%, and 29%, and a local recurrence rate of 66.7% at a median follow-up of 11 months [18]. In this context, the present case—a giant HCC (11.8 × 12.3 cm on preoperative CT; 11 cm on pathological examination) resected in an 89-year-old frail patient—falls at the upper boundary of the giant category and represents a surgically demanding scenario for which comparative data across open, laparoscopic, and robotic approaches in this specific patient group remain scarce.

In this case, a left lateral sectionectomy was performed via an open approach on 4 September 2025. Although minimally invasive approaches offer well-documented perioperative advantages in elderly patients, open resection was chosen on the basis of tumour size (11.8 × 12.3 cm), anatomical extent of hepatic involvement, and available institutional expertise. For tumours of this magnitude combined with the patient’s overall risk profile, open surgery was judged the safest option—a position consistent with the view that minimally invasive techniques should not be applied uniformly irrespective of tumour complexity.

### 3.1. Age-Based Approach

Patient age is one of the most common factors considered in scientific research. Many guidelines are based on age, and most studies use age as an inclusion or exclusion criterion. Due to the increasing number of liver cancer cases and the ageing population, many studies have attempted to stratify patients for liver resection based on age.

In a study of 150 individuals equally divided into two age groups, above and under 70 years, no difference was found in the rates of severe complications between these groups. Nineteen percent of patients in the elderly group were discharged to rehabilitation facilities, compared with one percent in the non-elderly group (*p* = 0.001). After univariate analysis, it was concluded that preoperative systemic chemotherapy and longer operative time were associated with higher morbidity in the elderly group. The study concluded that liver resection in the elderly is as safe as in younger patients [19].

The study by Yamoto et al. compared postoperative complications and survival at a single institution in 367 patients divided into three groups: 70–79, 80–84, and ≥85 years. Significantly more laparoscopic liver resections were performed in the ≥85 age group compared with the 70–79-year-old group. The authors cited lower intraoperative blood loss, shorter hospital stay, and lower postoperative morbidity as reasons for this. In this study, hypertension was the most common comorbidity in each group. The prognosis of patients in the ≥85 group was not significantly different from that in the other two groups. There was also no statistically significant difference in postoperative complications [4]. In addition, HCC-related or liver-related deaths in elderly patients were nearly identical to those in non-elderly patients. An association between age and non-liver-related death was observed. Predictive factors for patient prognosis in HCC included vascular invasion, tumour size, pTNM stage, AFP level, and the Geriatric 8 score. Patient age was not included. The authors concluded that age is not an absolute contraindication to liver resection [4]. A retrospective study published in 2024 reached a similar conclusion, stating that liver resection in selected elderly HCC patients can be performed safely. In this study, 364 patients were divided into three groups: less than 60 years, between 60 and 69 years, and above 70 years [20].

In a study evaluating early postoperative outcomes after liver resection—including duration of intensive care unit stay, length of hospital stay, occurrence of postoperative complications, and in-hospital mortality—in 460 patients divided into two groups (60 years or younger and 70 years or older; patients aged 61 to 69 years were not included in the study design), it was shown that age did not influence mortality and that the regenerative capacity of elderly livers is comparable to that of younger ones. A statistically significant difference was observed in the median hospital stay: 7 days for the younger group and 8 days for the elderly group (*p* = 0.007), and in the incidence of pneumonia—eight percent in the group aged 70 or older versus two percent in the group aged 60 or younger (*p* = 0.015). There was no statistically significant difference in the median duration of ICU stay or in liver-related complications [21].

In contrast to the previously mentioned studies, Riediger et al. showed that liver resections in patients aged 70 years or older are associated with a worse postoperative course than in the group younger than 70 years. They suggested that parenchyma-sparing resections should be preferred in these patients. The study included 703 patients. In multivariate analysis, age above 70 years was an independent risk factor for postoperative morbidity, but not for postoperative mortality or reoperation. Independent risk factors for worse postoperative outcomes in the whole cohort were major resections, longer operating time, need for intraoperative transfusions of blood products, as well as biliodigestive anastomosis (BDA) and inferior vena cava (IVC) resection [22]. A nationwide study in the Netherlands involving 6587 individuals divided into three groups—those younger than 70, those between 70 and 80 years (septuagenarians), and those 80 years or older (octogenarians)—showed higher 30-day morbidity and mortality in elderly patients, mainly due to non-surgical cardiopulmonary complications. Thirty-day major morbidity was 11% in septuagenarians and 12% in octogenarians, compared to 9% in younger patients (*p* = 0.049). Higher 30-day mortality was observed in septuagenarians (4%) and octogenarians (4%) compared to younger patients (2%, *p* < 0.001). The main reason for this was the more frequent occurrence of non-surgical cardiopulmonary complications. There were no differences in liver-specific complications between the groups. Length of stay was also longer in septuagenarians and octogenarians compared to the group younger than 70 years [23].

A meta-analysis published in 2024 by Lee et al. included 56 studies: 47 from Asia and nine from Europe. Only patients with HCC were included, divided into non-elderly and elderly groups. The age cut-off for each group was specified in every study. In the analysed sample, there was no statistically significant difference in overall survival or disease-free survival between the non-elderly and elderly groups. These results suggest that factors other than surgery influence overall survival and disease-free survival, such as increased lifespan and improved performance in the elderly [24,25].

Currently, selection criteria for patients undergoing surgery enable the identification of only well-conditioned patients. Others can be treated with local ablative therapies, transarterial therapies, systemic therapies, or radiation therapy [26]. Surgical techniques and postoperative management are also more advanced than previous methods, which may contribute to good outcomes in the elderly group [27]. The region where patients originate also appears to be important. The difference in overall survival and disease-free survival between elderly and non-elderly groups is less prominent in Asia compared to non-Asian regions, most likely because the aetiology of HCC in Asia is related to viral hepatitis. The difference between the average age of non-alcoholic fatty liver disease-associated HCC patients and viral hepatitis-associated HCC patients is about 5 to 10 years [28,29,30].

Therefore, a lower median age in the Asian region could account for the less pronounced difference in overall survival (OS) and disease-free survival (DFS) between elderly and non-elderly groups. There is no statistical difference in overall postoperative mortality; however, 30-day, 90-day, and in-hospital mortality rates are higher in the elderly group [31]. Another meta-analysis of 66 studies showed higher 30-day and 90-day mortality in the ≥70, ≥75, and ≥80 age groups compared to the ≥65 group. As in previous studies, it was concluded that age alone should not be used as an exclusion criterion [32]. A meta-analysis of 27 studies found that the elderly subgroup with colorectal liver metastasis had significantly lower overall survival than the non-elderly subgroup. There was also significantly higher 30-day mortality in the elderly CLM subgroup (*p* < 0.00002). This difference was not observed in the subgroup with HCC, suggesting that selection criteria should consider tumour type and progression in addition to age and functional status [33].

The postoperative course of our patient was complicated by transient haemodynamic instability and respiratory insufficiency requiring brief ICU support, with discharge on postoperative day six. This reflects the type of manageable non-surgical complications consistently reported in studies on liver resection in elderly patients. Importantly, no liver-specific surgical complications occurred. This outcome supports the argument that in carefully selected elderly patients with adequate preoperative optimisation, perioperative risk, although elevated, remains within an acceptable and manageable range.

### 3.2. Frailty-Based Approach

Frailty is characterised by deficits across various health domains, including physical, cognitive, psychological, and social aspects. It is estimated to affect more than 10% of elderly patients in England, with prevalence rising steeply with age [34,35].

Frailty alone is not a clinical entity, as frail patients do not exhibit a consistent pattern of common characteristics. The aetiology and pathophysiological mechanisms can also differ significantly among patients identified as frail [34]. There are different approaches to assessing frailty.

The modified frailty index (mFI) is an abbreviated 11-variable version of the Canadian Study of Health and Aging Frailty Index, which contains 70 variables. This method of frailty assessment represents the accumulating deficits model of frailty [36]. The mFI-5, a further abbreviated version, consists of five items: diabetes mellitus, increased blood pressure requiring medication, non-independent functional status, respiratory pathology (history of COPD or pneumonia), and congestive heart failure within 30 days of surgery. The mFI-5 is a predictive index used to estimate the likelihood of surgical complications preoperatively [37].

Another method is the geriatric assessment score, which provides a comprehensive assessment of an individual’s frailty status. Geriatric assessment includes evaluation of the patient’s physical, functional, social, and psychological well-being [38].

In a single-centre, observational case–control study, frail patients had a higher rate of morbidity (*p* = 0.04) compared to non-frail patients. Frail patients who underwent major liver resections had a higher probability of post-hepatectomy liver failure (*p* = 0.009), postoperative bleeding (*p* = 0.002), and a longer ICU stay (*p* = 0.003) compared to non-frail patients. There was no difference in mortality or length of stay. The study used the mFI-5 to quantify frailty, considering patients with an mFI-5 score of 1 or more as frail [37].

Frail patients are not contraindicated for surgery. Frailty predicts the probability of postoperative complications. A higher frailty score should predict worse outcomes, which could help patients and surgeons make therapy plans. In a meta-analysis of 10 studies including 71,102 patients, grouped into non-frail (53,928 patients) and frail (17,167) categories, the scores used for frailty assessment were the 11-factor modified frailty index, the 5-factor modified frailty index, the clinical frailty scale, the Johns Hopkins frailty assessment calculator, and the Kihon checklist. In the frail group, a higher incidence of postoperative liver failure (*p* < 0.001), mortality (*p* < 0.001), and readmission rate (*p* = 0.021) was observed. Preoperative albumin was also lower in the frail group, consistent with the malnutritional status of frail patients [39].

A meta-analysis of 20 studies described several strategies for patient risk stratification [40]. One of these is sarcopenia, which may be a good predictive factor for postoperative mortality and the development of postoperative fistulas. Sarcopenia is defined by the total psoas area measured on CT scan or by psoas density [41,42,43]. In the study by Gani, the revised Frailty Index, combined with ASA class, BMI, serum albumin, haematocrit, underlying pathology, and type of liver resection, was shown to predict postoperative morbidity, mortality, and prolonged hospital stay [44]. The Modified Frailty Index was validated in over 13,000 patients. The risk of mortality increased 2–10 times, and grade 4 complications increased 2–6 times for each point on the mFI [45]. Chen et al. showed in 1928 patients who underwent pancreatic and liver surgery that a score of two or more on the five-item mFI is associated with an increased risk of postoperative complications [46].

Maegawa et al. demonstrated in a retrospective analysis that patients with higher frailty scores experienced more Clavien–Dindo grade IV complications and higher 30-day mortality. In this study, 24,150 hepatectomies were reviewed: 3849 were laparoscopic, 529 were robotic, and 19,772 were open procedures. The study used a five-variable modified frailty index to categorise patients. The most common indication for surgery was metastatic disease (47.8%), while 17% of cases were for benign tumours. The incidence of Clavien–Dindo grade IV complications increased with higher mFI scores (incidence for mFI 0, 1, 2, 3, 4 was 3.9%, 6.3%, 10%, 8.1%, and 50%, respectively; *p* < 0.001). A similar trend was observed in 30-day mortality (incidence for mFI 0, 1, 2, 3, 4 was 0.9%, 1.9%, 3.3%, 1%, and 40%, respectively; *p* < 0.001). There was also a difference in the occurrence of Clavien–Dindo grade IV complications between minimally invasive and open hepatectomies in frail patients (Open: 7.7%; Laparoscopic: 3%; Robotic: 2.3%; *p* < 0.001) as well as in non-frail patients (Open: 4.6%; Laparoscopic: 1%; Robotic: 2.6%; *p* < 0.001). Postoperative mortality was also higher in frail patients undergoing open hepatectomies (Open: 2.4%; Laparoscopic: 0.8%; Robotic: 0.4%; *p* < 0.001) and in non-frail patients (Open: 1%; Laparoscopic: 0.1%; Robotic: 1.1%; *p* < 0.001). In the subgroup analysis, patients who underwent converted operations had similar rates of Clavien–Dindo grade IV complications to those who had an open approach. In the frail group, there was a significantly higher incidence of post-hepatectomy liver failure (grades A–C: 5.4% vs. 4.1%; *p* < 0.001) compared to the non-frail group. There was no significant difference between frail and non-frail patients in the incidence of bile leak [47].

The Albumin–Bilirubin score (ALBI) was developed as a predictive factor for post-hepatectomy survival in patients with hepatocellular carcinoma [48]. This study showed that incorporating functional status (mFI) with the Albumin–Bilirubin score improves the discriminative capacity for predicting Clavien–Dindo grade IV complications and 30-day mortality compared to ALBI alone. The combination of mFI and ALBI improved the AUC from 0.609 to 0.647 (*p* < 0.001) for Clavien–Dindo grade IV complications and from 0.663 to 0.72 (*p* < 0.001) for 30-day mortality [47].

The frailty scores obtained in this patient illustrate how these tools can inform preoperative decision-making in practice. An mFI-5 score of 3 placed her firmly in the high-risk frail category; according to Maegawa et al., this threshold carries a substantially increased risk of Clavien–Dindo grade IV complications and 30-day mortality. The G8 score of 6 out of 17—well below the impairment cut-off of 14—reflected severe weight loss (25 kg over six months), polypharmacy, and advanced age, and indicated the need for comprehensive geriatric assessment before any surgical intervention. Preoperative albumin of 30 g/L was consistent with the nutritional compromise captured by G8 screening, in line with the observation by Lunca et al. that frail patients undergoing liver resection have significantly lower preoperative albumin levels [39]. Taken together, these findings guided rather than precluded the surgical decision: they prompted targeted cardiac workup, which identified and treated haemodynamically significant LAD stenosis prior to resection. The subsequent postoperative course—haemodynamic instability, pleural effusion, and transient oxygen requirement—matched the risk profile predicted by these scores and confirmed the need for planned ICU-level monitoring in this patient group.

### 3.3. Heart Comorbidies and Liver Resection

Preoperative cardiac disease and postoperative cardiovascular complications place a significant burden on the healthcare system, leading to prolonged hospitalisation and increased medical costs, particularly during the perioperative period. Additionally, one-third of perioperative deaths are due to major perioperative cardiac complications [49]. Major liver surgery is especially challenging for patients with cardiac comorbidities, as maintaining a low central venous pressure requires strategies such as fluid restriction and the use of vasopressors [50].

In the retrospective study by Tran et al., patients who underwent major hepatectomy with cardiac comorbidity had a significantly higher incidence of postoperative cardiac arrest requiring cardiopulmonary resuscitation (*p* = 0.001) compared to those without cardiac comorbidity. The incidence of myocardial infarction was also significantly higher in the group with cardiac comorbidity (*p* = 0.011). Multivariate analysis did not show a significant association between cardiac comorbidity and 30-day mortality, but functional impairment, older age, and malnutrition were significant factors. Patients with a history of percutaneous coronary intervention (PCI) had a threefold increased risk of any cardiac complication. Previous studies have shown that the risk of complications is 1% six months after PCI [51].

Although patients with previous PCI within six months were excluded from this study, those with a history of PCI had a cardiac complication rate of 6.4% and a threefold increased risk of cardiac arrest. A limitation of the study was the lack of specification regarding the type of stent used (bare-metal stent or drug-eluting stent) [52].

In our patient, percutaneous revascularisation without stenting was chosen prior to liver resection to keep the required dual antiplatelet period as short as possible, thereby limiting perioperative bleeding risk while still addressing haemodynamically significant LAD stenosis. Despite this preparation, the patient developed postoperative haemodynamic instability requiring noradrenaline support, consistent with the findings of Tran et al., who reported a threefold increased risk of cardiac complications in patients with a prior PCI history. This highlights the need not only to address cardiac disease preoperatively, but to ensure ICU-level monitoring is planned from the outset in this patient group.

## 4. Conclusions

The occurrence of non-surgical postoperative complications, rather than liver-specific surgical complications, in our patient (such as haemodynamic instability and cardiopulmonary deterioration) aligns with the literature. Therefore, it is important to consider frailty, comorbidities, nutritional status, and functional reserve, rather than age alone, to accurately estimate a patient’s surgical risk. This is particularly relevant given the rapidly ageing global population and increasing life expectancy, where very elderly patients with multiple comorbidities represent a growing surgical cohort that remains largely under-represented in clinical research. Prospective multicentre studies specifically targeting this population are needed to inform evidence-based guidelines. Nevertheless, our case demonstrates that with thorough multidisciplinary evaluation and targeted preoperative optimisation, surgical resection may remain a viable option even in nonagenarians with significant comorbidity burden.

In elderly patients, there is no single clear-cut risk factor for liver resection. Instead, decisions should be based on clinical findings and the patient’s overall status. Frailty scores can help identify patients at higher risk of postoperative complications and those who would benefit most from preoperative optimisation therapy. Frailty is a strong negative predictor of postoperative outcomes and provides a better prediction model than age alone.

In addition to clinical tools, the multidisciplinary team plays a crucial role in patient selection. Not only does the surgery itself affect the complication rate, but so does the quality of preoperative and postoperative optimisation. The multidisciplinary team is essential in presenting realistic goals and future management plans to patients, which is extremely important in this group.

## Figures and Tables

**Figure 1:**
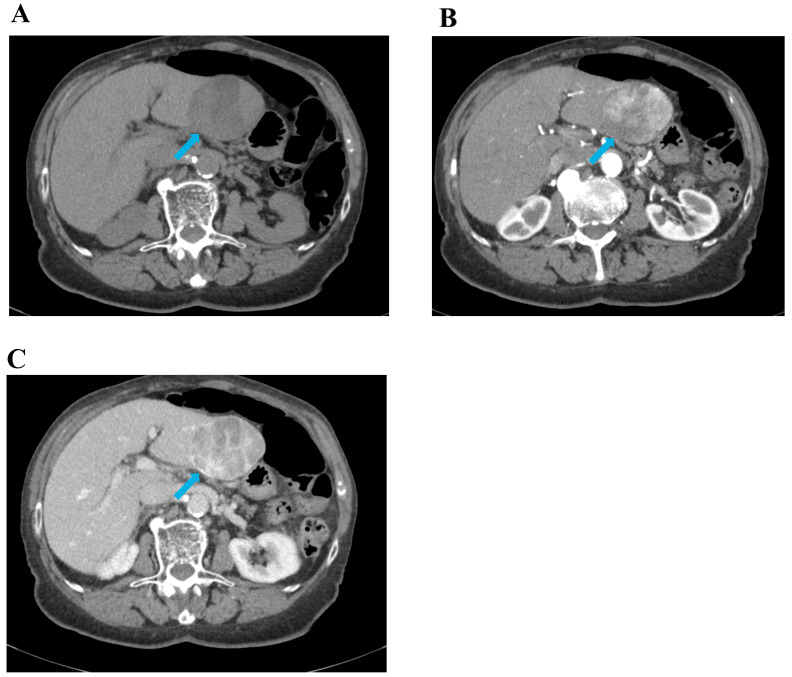
Abdominal CT in three phases, all in the axial plane. The blue arrow indicates a tumour in segment III of the liver, approximately 6.1 cm in diameter. Panel (**A**) shows the native (non-contrast) phase; panel (**B**) shows the arterial phase; panel (**C**) shows the venous phase of contrast enhancement.

**Figure 2:**
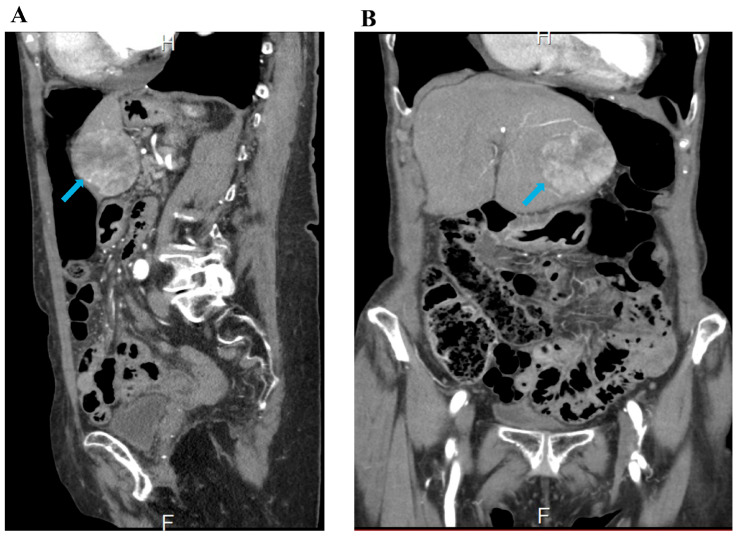
Contrast-enhanced CT of the abdomen in the arterial phase. The blue arrow indicates a tumour located in segment III of the liver, measuring 6.1 cm in diameter. Panel (**A**) shows the lesion in the sagittal plane; panel (**B**) shows the same lesion in the coronal plane.

**Figure 3:**
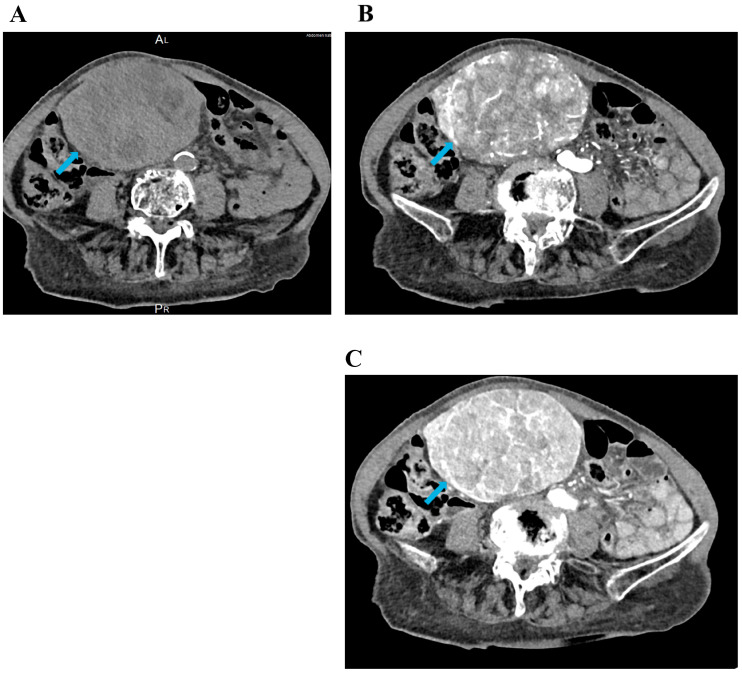
Abdominal CT in three phases, all in the axial plane. The blue arrow indicates a large tumour in segment III of the liver, measuring 11.8 × 12.3 cm, causing hepatomegaly with a liver diameter of 20 cm in the midclavicular line. Panel (**A**) represents the native (non-contrast) phase; panel (**B**) shows the arterial phase; panel (**C**) shows the venous phase of contrast enhancement.

**Figure 4:**
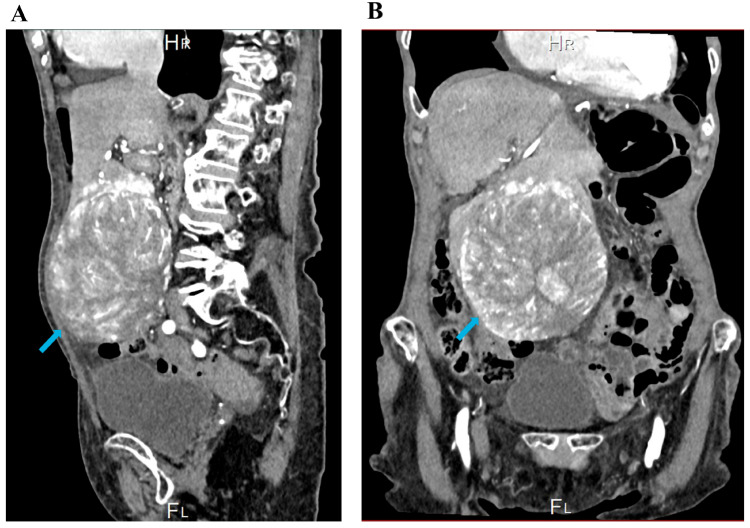
Contrast-enhanced CT of the abdomen in the arterial phase. The blue arrow indicates a large tumour located in segment III of the liver, measuring 11.8 × 12.3 cm, resulting in hepatomegaly with a liver diameter of 20 cm in the midclavicular line. Panel (**A**) shows the lesion in the sagittal plane; panel (**B**) shows the same lesion in the coronal plane.

**Figure 5:**
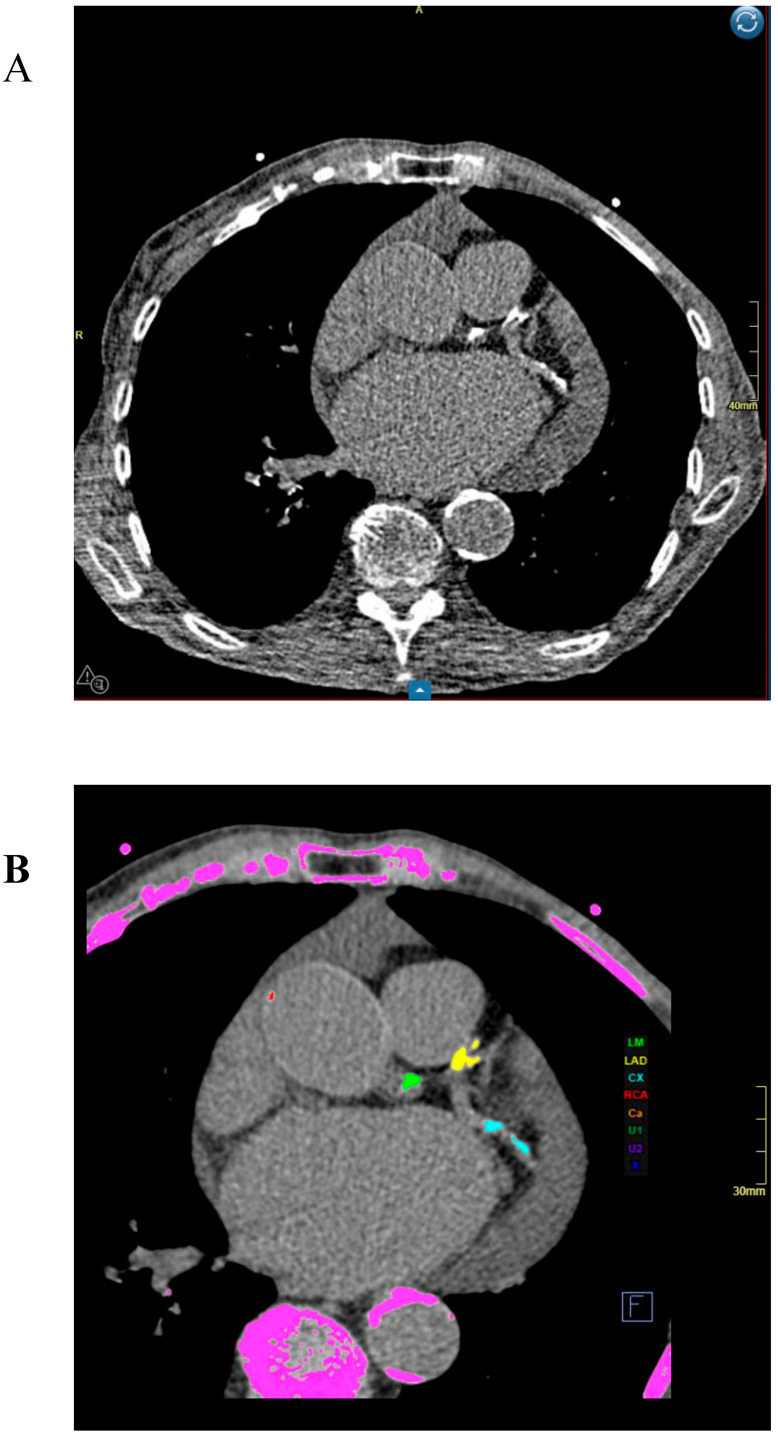
Cardiac CT (**A**) Cardiac CT was performed due to suspected angina pectoris at rest, revealing pronounced calcification of the coronary arteries. The extent of calcification caused artefacts that prevented reliable assessment of coronary artery narrowing on CT, necessitating subsequent coronary angiography. (**B**) Colour-coded calcium-scoring reconstruction at the same axial level, shown for anatomical orientation and to identify the coronary artery territories. The dedicated post-processing software assigns each calcified focus to its corresponding vessel and overlays it in a distinct colour: the left main (LM, green color), the left anterior descending artery (LAD, yellow color) and the circumflex artery (CX, cyan color) are indicated. This colour map illustrates only the distribution of coronary calcium and does not allow assessment of luminal stenosis.

**Figure 6:**
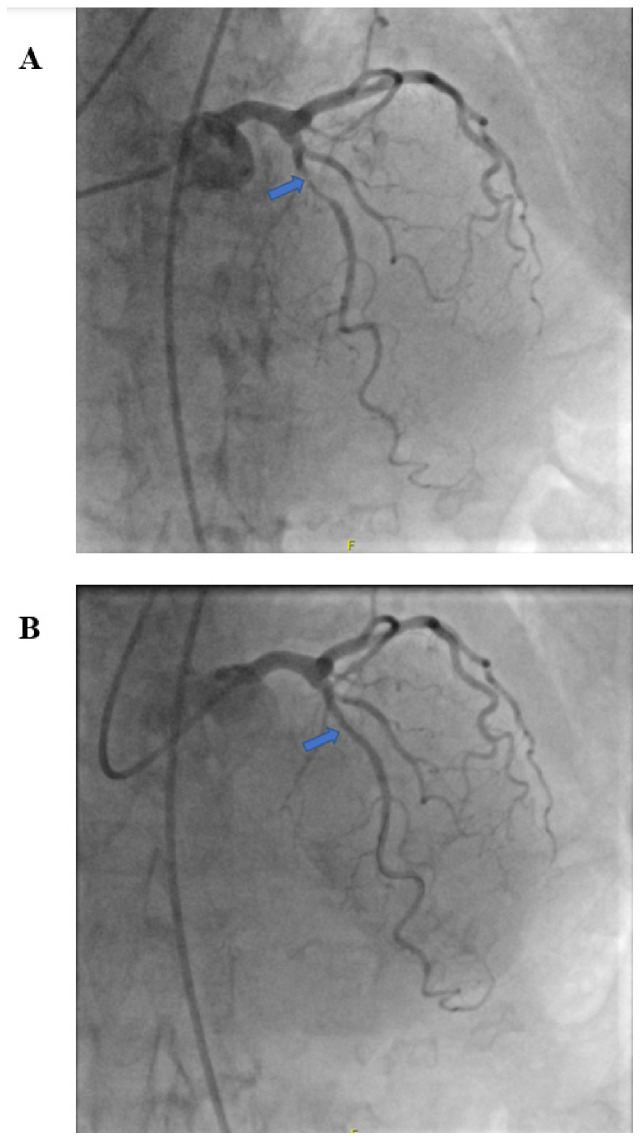
Coronary angiography shows haemodynamically significant narrowing of the proximal LAD. In panel (**A**), the blue arrow indicates the site of stenosis. Panel (**B**) shows the same location, indicated by the blue arrow, after percutaneous revascularisation performed without stenting. Borderline changes were noted in the remaining coronary vessels (LCA, LCX, and RCA).

**Table 1:** Overview of laboratory test results obtained at three time points: initial evaluation in May 2024, preoperative assessment in July 2025, and the early postoperative period following left lateral sectionectomy. Corresponding normal reference ranges are listed alongside each parameter.

Test	May 2024 Result	July 2025 Result	Post-Op Result	Normal Range
White blood cells (WBC)	6.3 × 10^9^/L		10.2 × 10^9^/L	4.0–10.0 × 10^9^/L
Red blood cells (RBC)	4.13 × 10^12^/L		3.32 × 10^12^/L	3.80–4.80 × 10^12^/L
Haematocrit (Hct)	0.39		0.307	0.360–0.460
Platelets (PLT)	197 × 10^9^/L		278 × 10^9^/L	150–410 × 10^9^/L
Haemoglobin	127 g/L	120 g/L	103 g/L	120–150 g/L
Glucose	5.8 mmol/L	5.7 mmol/L	5.1 mmol/L	3.6–6.1 mmol/L
Potassium (K)	4.1 mmol/L	4.8 mmol/L	4.4 mmol/L	3.8–5.5 mmol/L
Sodium (Na)	136 mmol/L	139 mmol/L	132 mmol/L	135–145 mmol/L
Urea	11.9 mmol/L	12.1 mmol/L	10.4 mmol/L	2.5–6.7 mmol/L
Creatinine (Cr)	126 µmol/L	149 µmol/L	81 µmol/L	49–90 µmol/L
eGFR	34 mL/min/1.73 m^2^	27 mL/min/1.73 m^2^	55 mL/min/1.73 m^2^	
NT-proBNP	1368 ng/L			
Chloride (Cl)		99 mmol/L	101 mmol/L	98–107 mmol/L
Total bilirubin (TBil)		12 µmol/L	34 µmol/L	3–22 µmol/L
Direct bilirubin (DBil)		6 µmol/L	22 µmol/L	Up to 7 µmol/L
Alkaline phosphatase (ALP)		1.85 µkat/L	6.17 µkat/L	0.55–1.64 μkat/L
Alpha-fetoprotein (AFP)	26 IU/mL	395.5 IU/mL		Up to 6.2 IU/mL
C-reactive protein (CRP)	5 mg/L	0.5 mg/L	124 mg/L	<5 mg/L
Procalcitonin (PCT)			0.24 µg/L	Up to 0.24 µg/L
Inorganic phosphate			1.19 mmol/L	0.74–1.52 mmol/L
Magnesium (Mg)			0.74 mmol/L	0.66–1.07 mmol/L
Albumin (Alb)			30 g/L	32–55 g/L
Total protein (TP)			55 g/L	64–83 g/L
INR			1.10	
Prothrombin time ratio (PTR)			0.80	
Alanine Aminotransferase (ALT)	0.3 µkat/L	1.17 µkat/L	5.56 µkat/L	up to 0.57 µkat/L
Aspartate Aminotransferase (AST)	0.37 µkat/L	1.16 µkat/L	3.69 µkat/L	up to 0.52 µkat/L
Gamma-glutamyl transferase (GGT)	1.52 µkat/L	2.65 µkat/L	8.87 µkat/L	up to 0.63 µkat/L
Alpha-amylase			0.46 µkat/L	0.52–1.78 µkat/L
Lipase (LIP)			0.16 µkat/L	Up to 1.07 µkat/L
Creatine kinase (CK)			0.61 µkat/L	Up to 2.41 μkat/L
Lactate dehydrogenase (LDH)			5.58 µkat/L	Up to 4.12 µkat/L

Note: Empty cells indicate that the parameter was not measured at that time point.

## Data Availability

The original contributions presented in this study are included in the article. Further inquiries can be directed to the corresponding author.
